# Multimodality imaging in the diagnostic management of concomitant aortic stenosis and transthyretin-related wild-type cardiac amyloidosis

**DOI:** 10.3389/fcvm.2023.1108696

**Published:** 2023-03-14

**Authors:** Angelica Cersosimo, Andrea Bonelli, Carlo M. Lombardi, Antonella Moreo, Matteo Pagnesi, Daniela Tomasoni, Gianmarco Arabia, Enrico Vizzardi, Marianna Adamo, Davide Farina, Marco Metra, Riccardo M. Inciardi

**Affiliations:** ^1^ ASST Spedali Civili di Brescia, Division of Cardiology and Department of Medical and Surgical Specialties, Radiological Sciences and Public Health, University of Brescia, Brescia, Italy; ^2^ ASST Grande Ospedale Metropolitano Niguarda, “A. De Gasperis” Department, Cardiology IV, Milan, Italy; ^3^ ASST Spedali Civili di Brescia, Division of Radiology and Department of Medical and Surgical Specialties, Radiological Sciences and Public Health, University of Brescia, Brescia, Italy

**Keywords:** cardiovascular imaging, aortic stenosis, cardiac amyloid, echocardiography, magnetic resonance imaging, scintigraphy

## Abstract

Severe aortic stenosis (AS) is the most common valvular heart disease with a prevalence rate of more than 4% in 75-year-old people or older. Similarly, cardiac amyloidosis (CA), especially “wild-type transthyretin” (wTTR), has shown a prevalence rate ranging from 22% to 25% in people older than 80 years. The detection of the concomitant presence of CA and AS is challenging primarily because of the similar type of changes in the left ventricle caused by AS and CA, which share some morphological characteristics. The aim of this review is to identify the imaging triggers in order to recognize occult wtATTR-CA in patients with AS, clarifying the crucial step of the diagnostic process. Multimodality imaging methods such as echocardiography, cardiac magnetic resonance, cardiac computed tomography, and DPD scintigraphy will be analyzed as part of the available diagnostic workup to identify wtATTR-CA early in patients with AS.

## Introduction

Severe aortic stenosis (AS) is the most common valvular heart disease with a prevalence rate of more than 4% in 75-year-old people or older (1). In postmortem studies (2, 3), cardiac amyloidosis (CA), especially “wild-type transthyretin” (ATTRwt), has shown a prevalence rate ranging from 22% to 25% in people older than 80 years, with a predominance for male patients. The detection of the concomitant presence of CA and AS is seeing an increase because of new available diagnostic tools. However, the diagnostic process is challenging primarily because of the similar type of changes in the left ventricle (LV) structure and function caused by AS and CA, which share some morphological characteristics (4). Diagnostic management of these conditions appears critical, as it has been shown that the association of AS and CA increases the risk of mortality, especially because both pathologies are associated with heart failure (HF) development (5–8).

**Figure 1:**
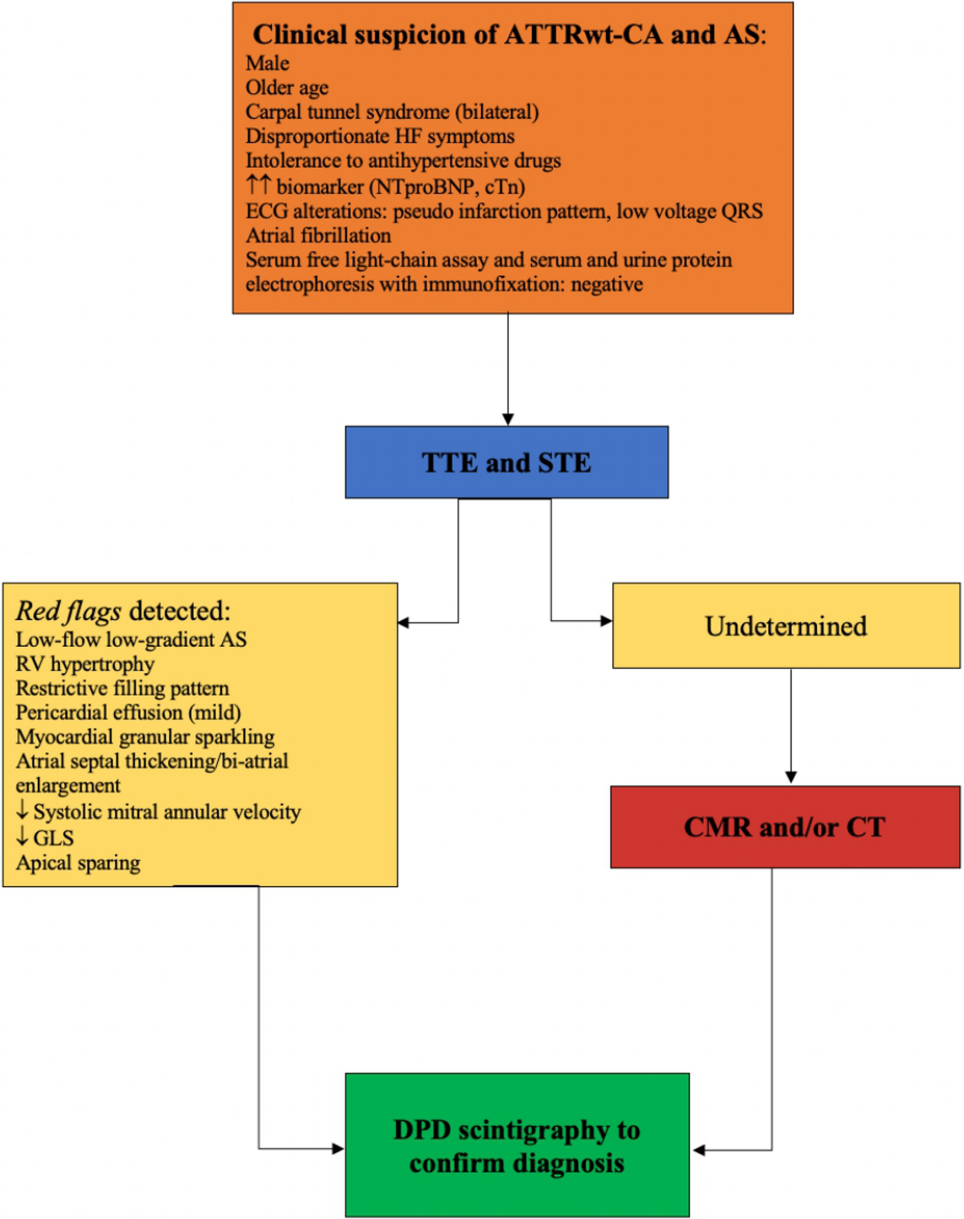
An imaging algorithm proposed to evaluate the presence of ATTRwt-CA in severe AS.

In a case series, ATTRwt-CA has been found in 6% of patients with AS aged >65 years undergoing surgical aortic valve replacement. Moreover, it has been associated with a mortality rate of 50% (9). The prevalence of occult ATTRwt-CA is even higher among patients undergoing trans-catheter aortic valve replacement (TAVR), settling at 16% and reaching 22% in males (10), probably because of the older age of the population. Similarly, in diagnosed ATTRwt-CA patients, the prevalence of moderate to severe AS was high, with a rate of 27% (11). On the other hand, the prevalence rate of moderate to severe AS is only 9% in people diagnosed with light chain (AL)-CA (12). In fact, the coexistence of ATTRwt-CA and AS is more common than that of AL-CA and AS because of the different ages of presentation and the natural history of the disease (9, 13). Indeed, AL-CA usually affects people in the sixth or seventh decade and has a poor prognosis, because it is the consequence of a hematological disorder (i.e., multiple myeloma) (12). Some clinical characteristics are suggestive of the presence of CA: bilateral carpal tunnel syndrome, disproportionate HF symptoms, and intolerance to antihypertensive drugs. Patients with CA usually show higher levels of cardiac biomarkers, such as N-terminal brain natriuretic peptide (NT-proBNP) and cardiac troponin (cTn), and specific alterations of the electrocardiogram (ECG) (Figure 1), including atrial fibrillation (AF) (14, 15).

The diagnosis of CA in AS is challenging, particularly when echocardiographic phenotypical abnormalities (such as LV size, function, mass, and stroke volume) are attributed to AS (16). Transthoracic echocardiography (TTE) remains the first diagnostic approach to detect those features, which helps raise clinical suspicion. Subsequently, second-level diagnostic examinations must be performed. Scintigraphy with bone tracers has become essential for definitive diagnosis. The aim of this review is to identify the imaging triggers in order to recognize occult ATTRwt-CA in patients with severe AS, clarifying the crucial step of the diagnostic process.

## Echocardiography

Echocardiographic evaluation is the gold standard for AS diagnosis (17) and TTE is the first imaging approach when concomitant ATTRwt-CA and AS are suspected. Nowadays, several specific parameters have been reported to be useful in the diagnosis of ATTRwt-CA patients. The most frequent TTE parameters are based on LV and right ventricular (RV) wall thickening, elevated left-sided filling pressures (measured by *E*/*e*′ ratio), restrictive filling pattern, reduced peak systolic tissue velocity, thickening of atrioventricular valves and atrial septum, biatrial dilation, and mild pericardial effusion (12). The typical finding of granular sparkling of myocardium is detected in the advanced stages of ATTRwt-CA (12–19). In contrast, the indices of diastolic dysfunction and increased wall thickness are encountered at the early stages. Most of these parameters are common findings in patients with AS, making the differential diagnosis challenging. However, patients with ATTRwt-CA and severe AS appear to have a higher LV wall thickness, LV mass, and grade of diastolic dysfunction compared with patients with only severe AS (5, 10) (Figure 1). Moreover, a lower stroke volume index (SVi) and reduced lateral and septal mitral annular tissue Doppler systolic velocities (*S*′) have been described in patients with AS and concomitant ATTRwt-CA (5, 10).

Although two-dimensional TTE with Doppler and Tissue Doppler imaging are the first steps to identify the presence of AS with concomitant ATTRwt-CA, it is often difficult to differentiate the impact of both conditions on myocardial function, making it necessary to integrate the diagnostic assessment with other methods. Speckle tracking echocardiography (STE) is crucial in the assessment of CA patients because of two typical features: reduced global longitudinal strain (GLS) and the apical sparing pattern (20, 21). In ATTRwt-CA, GLS, as a myocardial deformation index, is reduced in the early phases. In one study, reduced GLS values correlated to AS severity, amyloid myocardium infiltration, and fibrosis proliferation (22). GLS impairment in ATTRwt-CA has a characteristic pattern called “apical sparing,” which refers to the relative reduction of the myocardial deformation of the basal segments compared with the apex (23). The coexistence of severe AS and ATTRwt-CA is associated with an early decrease of GLS: a value of −14% is sensitive but not specific for identifying ATTRwt-CA in patients with AS [area under the curve (AUC) 0.75] (24). Moreover, the apical sparing pattern is both sensitive and specific for the diagnosis of ATTRwt-CA in patients with increased left ventricular wall thickness (25). Significantly, reduced LV longitudinal deformation is common in patients with concomitant ATTRwt-CA and AS, when compared with patients with only AS (4, 9). The pattern apical sparing in patients with both AS and ATTRwt-CA has good accuracy (sensitivity 88%, specificity 68%, AUC 0.73) to diagnose ATTRwt-CA in patients with severe AS. Since the presence of apical sparing is common in AS even in the absence of ATTRwt-CA, it should be considered in combination with other echocardiographic parameters to raise the suspicion of ATTRwt-CA disease (24). On the other hand, AS is responsible for elevated wall stress and increased afterload that may mask the presence of apical sparing (10, 26). Thus, the discriminatory capacity of STE in patients with both severe AS and ATTRwt-CA needs further investigation.

An identification of the echocardiographic predictors of ATTRwt-CA in patients with AS seems essential for achieving a better selection of patients for providing specific treatments. The best independent echocardiographic predictor of ATTRwt-CA in AS is mitral annular tissue Doppler (*S*′) velocity <6 cm/s, with 100% sensitivity and 57% specificity (AUC 0.95) (10). Other echocardiographic parameters are a higher left ventricular mass (5, 6, 9), higher left ventricular SVi and left atrial volume index (LAVi) (5), shorter deceleration time (<200 ms), higher *E*/*A* ratio, higher *E*/*e*′ ratio, and lower myocardial contraction fraction (MCF) (10, 27) (Table 1). MCF, obtained by the ratio of left ventricular stroke volume (SV) to myocardial volume (MV), has also been shown to be superior to EF for predicting mortality in patients with ATTRwt-CA (AUC of 0.83), because it is a volumetric measure of myocardial shortening strictly correlated with GLS (patients with AL CA have lower ECV values than those with ATTRwt-CA) (28). In fact, an MCF below 25% was independently associated with a significantly greater risk of death (29). In addition, only the SVi and LAVi are significantly associated with mortality in patients with both pathologies (Table 2).

**Table 1:** Imaging RED FLAGS for suspicion of ATTRwt-CA in severe AS patients.

Imaging RED FLAGS for suspicion of ATTRwt-CA in severe AS		
TTE	RM	CT
RV hypertrophy Elevated filling pressures Restrictive filling pattern Reduction of peak systolic tissue velocity Thickening of the atrioventricular valves and atrial septum Biatrial dilation Mild pericardial effusion Myocardial granular sparkling Reduction of GLS Apical sparing pattern	Increase in myocardial mass Increased ECV Increase in myocardial native T1 mapping Diffuse LGE	Increased ECV Increase in myocardial iodine concentration

**Table 2:** Echocardiographic predictors of all-cause mortality in patients with aortic stenosis and ATTRwt-CA.

Study	Cavalcante et al. (5)	Rosenblum et al. (7)	Treibel et al. (9)	Rubin et al. (29)
Mitral annular *S*′ (cm/s)	Not a predictor of all-cause mortality HR 0.77 (0.56–1.06, 95% CI, *p* .10)	Not a predictor of all-cause mortality HR 0.89 (0.77–1.04, 95% CI, *p* .150)	NA	NA
IVST (cm)	Not a predictor of all-cause mortality HR 3.14 (1.03–9.56, 95% CI, *p* .04)	Not a predictor of all-cause mortality HR 1.56 (0.94–2.57, 95% CI, *p* .083)	NA	NA
AV mean gradient (mmHg)	Not a predictor of all-cause mortality HR 0.98 (0.96-1.00 95% CI *p* .13)	NA	Not a predictor of all-cause mortality HR 0.96 (0.92–1.00 95% CI *p* .07)	NA
*E*/*e*′ ratio	Predictor of all-cause mortality HR 1.02 (1.006, 1.04 95% CI *p* .01)	NA	NA	NA
LV mass index (g/m^2^)	NA	NA	Predictor of all-cause mortality HR 1.03 (1.00–1.52 95% CI *p* .05)	NA
SVi (mL/m^2^)	Predictor of all-cause mortality HR 0.95 (0.92, 0.98 95% CI *p* .001)	Predictor of all-cause mortality HR 1.67 (1.00–2.79 95%CI *p* .049)	NA	NA
LAVi (mL/m^2^)	Predictor of all-cause mortality HR 1.04 (1.02, 1.06 95% CI *p* < .001)	NA	NA	NA
GLS	NA	NA	NA	NA
MCF (%)	NA	Predictor of all-cause mortality HR 0.98 (0.96–1.00 95% CI *p* .041)	NA	Predictor of all-cause mortality HR 5.4 (1.82–15.86, 95% CI *p* .0024)

AV, aortic valve; GLS, global longitudinal strain; IVST, interventricular septal thickness; LV, left ventricular; Svi, left ventricular stroke volume index; MCF, myocardial contraction fraction; DT, deceleration time; LAVi, left atrial volume index; NA, not available.

Furthermore, the coexistence of both AS and ATTRwt-CA frequently results in a low-flow low gradient or a paradoxically low-flow AS (10–16). In patients with low-flow low-gradient AS and one or more TTE red flags, in addition to the TTE evaluation of AS values, the concomitant presence of amyloidosis must be suspected, especially in male patients over 65 years old (10, 17, 21, 29) (Table 3).

**Table 3:** Evaluation of the echocardiographic parameters of lone aortic stenosis vs. aortic stenosis associated with cardiac amyloidosis.

Study	Cavalcante et al. (5)	Scully et al. (6)	Rosenblum et al. (7)	Castaño et al. (10)	Nitsche et al. (32)
Number of patients	113	109	204	151	191
Population (lone AS vs. AS/ATTRwt-CA)	104 vs. 9	93 vs. 16	177 vs. 27	127 vs. 24	175 vs. 16
Mitral annular *S*′ (cm/s) (lone AS vs. AS/ATTRwt-CA)	4.8 ± 1.7 vs. 1.8 ± 0.5 (*p* .008)	0.06 ± 0.01 vs. 0.05 ± 0.01 (*p* .08)	6.3 ± 1.6 vs. 4.5 ± 1.4 (*p* < .001)	6.6 ± 1.5 vs. 4.0 ± 1.1 (*p* < .0001)	NA
IVST (cm) (lone AS vs. AS/ATTRwt-CA)	1.3 ± 0.3 vs. 2.9 ± 1.0 (*p* < .001)	1.3 ± 0.2 vs. 1.4 ± 0.3 (*p* .002)	1.2 ± 0.3 vs. 1.4 ± 0.4 (*p* .001)	1.1 ± 0.2 vs. 1.3 ± 0.3 (*p* .007)	1.5 vs. 1.55 (*p* .183)
AV mean gradient (mmHg) (lone AS vs. AS/ATTRwt-CA)	31 ± 15 vs. 30 ± 14 (*p* 924)	42 ± 14 vs. 38 ± 12 (*p* .36)	41 ± 14 vs. 35 ± 13 (*p* .090)	41.1 ± 13.8 vs. 35.2 ± 13.9 (*p* .060)	47.5 vs. 35.0 (*p* .004)
AV Peak velocity (cm/s) (lone AS vs. AS/ATTRwt-CA)	NA	4.12 ± 0.63 vs. 4.02 ± 0.62 (*p* .55)	4.2 ± 0.7 vs 3.9 ± 0.7 (*p* .063)	4.3 ± 0.7 vs. 4.0 ± 0.7 (*p* .078)	NA
*E*/*e*′ ratio (lone AS vs. AS/ATTRwt-CA)	Septal 25 ± 18 vs. 33 ± 10 (*p* .281) Lateral 18 ± 11 vs. 19 ± 4 (*p* 942)	Lateral 17 ± 8 vs. 21 ± 15 (*p* .28)	19 ± 9 vs. 22 ± 9 (*p* .092)	16 vs. 19 (*p* .075)	NA
E/A ratio (lone AS vs. AS/ATTRwt-CA)	NA	0.8 vs. 1.4 (*p* .07)	1.4 ± 1.0 vs. 2.4 ± 1.5 (*p* < .001)	0.90 vs. 2.30 (*p* .001)	NA
Deceleration time (m/s) (lone AS vs. AS/ATTRwt-CA)	NA	234 ± 92 vs. 238 ± 80 (*p* .87)	247 ± 83 vs. 196 ± 73 (*p* .003)	257 vs. 176 (*p* < .0001)	212 vs. 199 (*p* .161)
LV mass index (g/m^2^) (lone AS vs. AS/ATTRwt-CA)	NA	113 ± 37 vs. 137 ± 31 (*p* .01)	106 ± 31 vs. 136 ± 47 (*p* < .0001)	97.9 ± 25.4 vs. 129.8 ± 43.6 (*p* .002)	135.0 vs. 159.0 (*p* .016)
SVi (mL/m^2^) (lone AS vs. AS/ATTRwt-CA)	37 ± 12 vs. 25 ± 7 (*p* .003)	38 ± 12 vs. 35 ± 9 (*p* .29)	35 ± 10 vs. 31 ± 11 (*p* .047)	35.7 ± 9.6 vs. 29.9 ± 10.5 (*p* .009)	46.6 vs. 27.4 (*p* < .001)
LAVi (mL/m^2^) (lone AS vs. AS/ATTRwt-CA)	40 ± 15 vs. 51 ± 13 (*p* .037)	NA	51 ± 20 vs. 54 ± 15 (*p* .281)	49.2 ± 17.2 vs. 55.5 ± 15.8 (*p* .108)	NA
LA dimension (cm) (lone AS vs. AS/ATTRwt-CA)	NA	4.0 ± 0.7 vs. 4.4 ± 0.6 (*p* .08)	4.4 ± 0.7 vs. 4.8 ± 0.7 (*p* .005)	4.4 ± 0.7 vs 5.0 ± 0.7 (*p* .002)	61.0 vs. 64.0 (*p* .215)
AVA (cm^2^) (lone AS vs. AS/ATTRwt-CA)	0.5 ± 0.2 vs. 0.4 ± 0.2 (*p* .047) indexed	0.71 ± 0.23 vs. 0.72 ± 0.21 (*p* .92)	0.76 ± 0.23 vs. 0.80 ± 0.15 (*p* .391)	0.77 ± 0.19 vs. 0.80 ± 0.16 (*p* .358)	0.6 vs. 0.6 (*p* .669)
GLS (lone AS vs. AS/ATTRwt-CA)	NA	−15 ± 7 vs. −16 ± 6 (*p* .62)	NA	−15.7 ± 4.3 vs. −12.4 ± 5.2 (*p* .007)	−16.9 vs. −13.8 (*p* .72)
MCF (%) (lone AS vs. AS/ATTRwt-CA)	NA	24.5 ± 8.4 vs. 19.4 ± 7.2 (*p* .002)	37 ± 15 vs. 25 ± 11 (*p* < .001)	41.0 ± 15.5 vs. 26.4 ± 10.1 (*p* < .0001)	21.9 vs. 15.1 (*p* .001)
LVEF (%) (lone AS vs. AS/ATTRwt-CA)	NA	54 ± 10 vs. 58 ± 170 (*p* .18)	55 ± 15 vs. 48 ± 17 (*p* .026)	56.1 ± 14.1 vs. 47.6 ± 17.6 (*p* .011)	62.0 vs. 62.0 (*p* .576)

AV, aortic valve; GLS, global longitudinal strain; IVST, interventricular septal thickness; LV, left ventricular; SVi, left ventricular stroke volume index; MCF, myocardial contraction fraction; LAVi, left atrial volume index; LVEF, left ventricular ejection fraction; LAVi, left atrial volume index; LA, left atrial; AVA, aortic valve area; NA, not available.

Since echocardiographic parameters are crucial to predict the presence of cardiac amyloidosis, several risk scores have been developed, including the AL score and the IWT score by Boldrini et al. (20) and the AMYLY score by Aimo et al. (30). In addition, Vergaro et al. (31) showed that cardiac biomarkers (NT-proBNP <180 ng/L or hs-TnT <14 ng/L,) can refine the echocardiographic scores in patients with suspected CA and hematologic disease or an increased wall thickness.

Although available echocardiographic parameters may be useful in the clinical identification of CA (20), further investigations are needed to detect patients with concomitant conditions early by using standard and advanced echocardiography in clinical practice.

## Cardiac magnetic resonance

Cardiac magnetic resonance (CMR) has a sensitivity of 93% for diagnosing ATTRwt-CA (32–34). Moreover, late gadolinium enhancement (LGE) and extracellular volume (ECV) play an important role in evaluating ATTRwt-CA interstitial deposition. The most frequent LGE patterns in CA are subendocardial and global transmural. However, there are a number of patients with heterogeneous patterns for which it is not possible to correctly distinguish the etiology based only on LGE distribution. Indeed, CMR can increase the suspicion of disease if typical findings of CA are present, but it cannot achieve a definite diagnosis nor can differentiate the amyloid type (i.e., AL vs. ATTR) (35–37).

In ATTRwt-CA, transmural LGE involvement of the RV (increased wall thickness) is found in 37%–97% of patients, while increased thickness of the left atrial wall is seen in 70%–90% (34, 38, 39). In the setting of AS, focal myocardial fibrosis is a frequent finding with a characteristic mid-wall scar pattern, which is an independent predictor of death and is associated with increased myocardial injury and diastolic and systolic dysfunction (40, 41). Moreover, CMR planimetry of the aortic valve area (AVA) may be used when there are some limitations for routine TTE, especially in those with low-flow low-gradient AS.

However, AS-ATTRwt-CA patients may present different combinations of the CMR-LGE pattern (e.g., nonischemic or mid-wall LGE), with a sensitivity of 25%, especially in the early stages, causing difficulties in ATTRwt-CA diagnosis (32–34, 42–45). In contrast, elevated native myocardial T1 mapping and ECV in ATTRwt-CA and severe AS are more sensitive than LGE imaging, also showing good diagnostic accuracy in the early stages (34, 45, 46).

Cavalcante et al. (5) assessed native T1 mapping and ECV values in patients with dual pathology (AS-ATTRwt-CA) and showed a higher native T1 and ECV values in patients with AS-ATTRwt-CA rather than in lone AS ones (mean ECV 41.2% ± 16.7 vs. 27.9% ± 4.1, *p* < .001; mean native T1 1,125 ms ± 49 vs. 1,035 ms ± 60, *p* .002). Subsequently, Nitsche et al. (32) confirmed the low sensitivity of distinctive LGE patterns in patients with combined clinical entities and showed that ECV increased the CMR power of discrimination (0.756 AUC) to differentiate AS from AS-ATTRwt-CA (Table 4).

**Table 4:** CMR predictor parameters of concomitant aortic stenosis–cardiac amyloidosis.

Study (first author)	Cavalcante et al (5)	Treibel et al (9)	Nitsche et al (32)
ECV (%)	Higher in concomitant aortic stenosis and cardiac amyloidosis (Only AS 27.9 ± 4.1 vs. AS + wATTR-CA 41.2 ± 16.7) ***p* < .001**	N A	Higher in concomitant aortic stenosis and cardiac amyloidosis [only AS 26.7 (24.6–29.0) vs. AS + wATTR-CA 30.3 (28.1–33.5)] ***p* .003**
T1 mapping (ms)	Higher in concomitant aortic stenosis and cardiac amyloidosis (Only AS 1,035 ± 60 vs. AS + wATTR-CA 1,125 ± 49) ***p* .002**	NA	No significant differences [only AS 1,033 (1,008–1,063) vs. AS + wATTR-CA 1,051 (,1013–1,080)] *p* .196
LV mass index (g/m^2^)	Higher in concomitant aortic stenosis and cardiac amyloidosis (Only AS 73 ± 21 vs. AS + wATTR-CA 105 ± 21) ***p* < .0001**	NA	No significant differences [only AS 79.4 (63.3–90.2) vs. AS + wATTR-CA 93.9 (61.3–100.5)] *p* .163
LA area (cm^2^)	NA	NA	No significant differences [only AS 29.0 (26.0–35.0) vs. AS + wATTR-CA 31.0 (23.0–34.0)] *p* .854
RVEF (%)	NA	NA	No significant differences [only AS 55.0 (44.6–63.0) vs. AS + wATTR-CA 48.0 (36.0–63.0)] *p* .271

ECV, extracellular volume; LV, left ventricular; RVEF, right ventricular ejection fraction; LA, left atrial; NA, not available.

The bold significant predictors of concomitant presence of cardiac amiloydosis and aortic stenosis.

Given the high cost, the availability of CMR seems to impact its ability to act as a suitable screening tool in patients referred for severe AS, although it has proved to be an emerging tool for the diagnosis and prognosis of AS-ATTRwt-CA patients. However, contrast-enhanced CMR with T1 mapping and ECV should be recommended as the second step (after echocardiography with deformation analysis) to screen patients with severe AS when there is a suspicion of concomitant ATTRwt-CA (Figure 1).

## Cardiac computed tomography

There is limited evidence on the role of cardiac computed tomography (CT) in the diagnosis of ATTRwt-CA. Several studies have demonstrated a relationship between myocardial attenuation and iodine administration in patients with ATTRwt-CA, namely myocardial iodine concentration, providing a quantitative measure of total myocardial involvement (47). Moreover, myocardial iodine concentration accurately distinguishes ATTRwt-CA from other cardiomyopathies with a sensitivity of 100% and specificity of 92% (48). In fact, it has been shown that myocardial ECV assessed by means of CT well correlates with ATTRwt-CA because it reflects the myocardial interstitium (49–54). ECV assessment during CT evaluation in patients with AS undergoing TAVR has been recently demonstrated to reliably detect AS-CA with an AUC of 0.87. In addition, the measured ECV was able to track the degree of infiltration (54). From the practical point of view, additional acquisitions should be made for the calculation of the ECV, whereas a standard CT protocol for AS evaluation in patients undergoing TAVR does not provide such information. In fact, the formula for ECV quantification is complex and CT imaging requires a specific protocol with a dedicated software (51, 54).

Although preprocedural CT is routinely used for the treatment workup in patients undergoing TAVR, its diagnostic value to identify patients with concomitant AS-ATTRwt-CA has been poorly explored, warranting further studies. Myocardial ECV during routine CT pre-TAVR has been proposed as a possible tool to identify AS-ATTRwt-CA since it seems strictly related to the degree of infiltration (54) (Figure 1).

## Bone scintigraphy

The first diagnostic algorithm for ATTR-CA was proposed and validated in 2016 (55). Then, the 2019 expert consensus stated that endomyocardial or extracardiac biopsy may be avoided if bone scintigraphy is available, after excluding the presence of monoclonal protein (56). Three technetium-labeled radiotracers have been evaluated for ATTR-CA identification: Tc-99m-pyrophosphate (PYP), Tc-99m-3,3-diphosphono-1,2-propanodicarboxylic acid (DPD), and Tc-99m_hydroxymethylene diphosphonate (HMDP) (57). Moreover, molecular imaging with targeted amyloid-binding positron emission tomography radiotracers [11C-Pittsburgh compound B (11C-PIB), 18F-florbetapir, and 18F-florbetaben] is an emerging diagnostic approach that may distinguish cardiac amyloidosis from other forms of heart disease (58–60). A grade 2 or 3 uptake on 99mTc-3,3-diphosphono-1,2-propanodicarboxylic acid scintigraphy (Grade 0 = no uptake, Grade 3 cardiac uptake above rib level), using the Perugini score (61) with the concomitant absence of a monoclonal protein, has specificity and a positive predictive value of 100% for ATTR-CA (56). Cardiac uptake on bone scintigraphy, in fact, reflects the extent of ATTR-CA infiltration and correlates with echocardiography and laboratory alterations (61–64). Therefore, this imaging method is safer than cardiac biopsy for exclusion of ATTR-CA with a greater sensitivity in diagnosing occult ATTR-CA than CMR (9). Several studies (57, 64, 65) have demonstrated a strong correlation between cardiac uptake, cTn, and LV mass index despite LV mean wall thickness. However, genotyping is required to distinguish ATTRwt-CA from hereditary ATTR-CA (4).

Treibel et al. (9) showed that postoperative bone scintigraphy was diagnostic for ATTRwt-CA (Grade 2 cardiac uptake) in patients with severe AS undergoing aortic valve replacement, in agreement with the positivity of intraoperative biopsies. This allowed to identify a 6% prevalence of ATTRwt-CA in patients with severe AS. Instead, a prevalence of 16% of ATTRwt-CA in patients with severe AS (especially with the phenotype low-flow low gradient with a mildly reduced ejection fraction) undergoing TAVR was highlighted by Castaño et al. (10). In addition, Longhi et al. (16) detected the presence of ATTRwt-CA through bone scintigraphy on five of 43 patients with severe AS, which was also confirmed by endomyocardial biopsy.

Therefore, when echocardiography reveals a severe AS and one or more red flags for ATTRwt-CA (19, 20), or a low-flow low-gradient AS in elderly patients, DPD scintigraphy should be performed to rule out the suspicion of concomitant ATTR-CA (Figure 1). Bone scintigraphy provides additional information on elderly patients with severe AS and gives us the discretion of choosing the appropriate treatment to increase survival rates. Moreover, targeting patients with severe AS, especially those with low-flow low-gradient AS with preserved EF, allows an early diagnosis of ATTR-CA and timely treatment, so as to significantly reduce mortality and to guide treatment decision-making of AS (11).

## Conclusion

The rate of the concomitant presence of ATTRwt-CA and severe AS is increasing, especially in the elderly population, in terms of the age-dependent penetrance of both conditions. When clinical characteristics and noninvasive imaging (i.e., TTE) suggest ATTRwt-CA in severe AS, it is important to perform an investigation using newer and combined imaging methods. The best diagnostic workup in severe AS with ATTRwt-CA remains a matter of debate. Indeed, several authors did not exclusively include ATTRwt-CA, but frequently AL and ATTR-CA, potentially biasing the interpretation of the results. This should be at least acknowledged as a limitation of this review.

However, to obtain maximal benefit from new and emerging therapies for ATTRwt-CA, an identification of concomitant conditions in the early stage of the disease should be the first goal. The issue whether ATTRwt-CA treatment should be initiated before or after approaching the valvular pathology needs further examination. Currently, available cardiovascular imaging tools should be routinely used to raise the suspicion of concomitant ATTRwt-CA-AS for an appropriate and precise diagnostic management of this condition.

The coexistence of ATTRwt-CA and severe AS in a patient has important implications for diagnosis, management, and prognosis. Screening for ATTRwt-CA in these patients is mandatory for tracking the growing prevalence of ATTRwt-CA [the median prevalence of AS and cardiac amyloidosis of 8% (CI, 5%–13%)] (66). For these purposes, a target therapy for ATTRwt-CA needs to be formulated before treatment is initiated for severe AS. Moreover, the treatment of AS improves survival rates even in ATTRwt-CA patients. Transcatheter aortic valve replacement may be preferred to surgery, given the high surgical risk and the fragility of the amyloid infiltrated tissue (4).

In clinical practice, when we approach an elderly patient with AS, especially if is a low-flow low-gradient one, we have to raise a suspicion for ATTRwt-CA after performing a careful clinical evaluation (bilateral carpal tunnel syndrome, hypotension or normotensive if previous hypertensive ECG pseudoinfarct pattern or low/decreased QRS voltage to the degree of LV thickness or AV conduction disease, disproportionally elevated NT-proBNP to the degree of HF, persisting elevated troponin levels) (67). However, the role of imaging is crucial for the screening, diagnosis, and staging of amyloidosis.

TTE is undoubtedly the first imaging method that raises the suspicion of amyloidosis in patients with AS. Through TTE, we evaluate the presence of AS and one or more of the following characteristics: granular sparkling of the myocardium, increased right ventricular wall and valve thickness, pericardial effusion, RV hypertrophy, elevated filling pressures and/or restrictive filling pattern, reduction of peak systolic tissue velocity, thickening of the atrioventricular valves and atrial septum, biatrial dilation, reduction of GLS, and apical sparing pattern. If one or more of the aforementioned echocardiographic features is present concomitant with AS (14), the second step is bone scintigraphy. However, there is no evidence on the use of bone tracers to stage cardiac amyloidosis, and the Perugini grade has not been shown to correlate with survival. Yet, bone scintigraphy (accompanied by hematologic tests) has been shown to play an increasingly high diagnostic role.

Subsequently, if echocardiographic parameters are undetermined, a second-level imaging evaluation can be performed, which is the CMR. An increased myocardial mass, ECV, native T1 mapping, and diffuse LGE allow to identify the amyloid disease. CMR may be used to identify typical features of cardiac amyloidosis, supporting efforts aimed at performing endomyocardial biopsy in the presence of a monoclonal protein and no abnormal cardiac uptake of bone tracers, especially when a histological analysis of extracardiac tissues fails to detect amyloid deposits.

CT is used routinely as a preprocedure in all patients undergoing TAVR; although some features may suggest the presence of concomitant cardiac amyloidosis (the presence of increased ECV and increased myocardial iodine concentration), its role in diagnosing it has not been validated. Therefore, although CT is a secondary imaging method, it must always be accompanied by CMR or bone scintigraphy for diagnosing amyloidosis.

It is important to take into consideration the fact that even if the ultimate goal of screening is to detect ATTRwt-CA, hematologic testing to rule out AL-CA and genetic testing to rule out ATTRv-CA are mandatory.

In conclusion, in patients with the concomitant presence of ATTRwt-CA and severe AS, a multidisciplinary assessment to evaluate the best suitable treatment approach is necessary. Accordingly, a multimodality imaging screening for amyloidosis should be performed in all elderly patients with AS in order to choose the best treatment strategy and to significantly reduce mortality.
